# PD-L1 Expression in Systemic Immune Cell Populations as a Potential Predictive Biomarker of Responses to PD-L1/PD-1 Blockade Therapy in Lung Cancer

**DOI:** 10.3390/ijms20071631

**Published:** 2019-04-02

**Authors:** Ana Bocanegra, Gonzalo Fernandez-Hinojal, Miren Zuazo-Ibarra, Hugo Arasanz, Maria Jesus Garcia-Granda, Carlos Hernandez, Maria Ibañez, Berta Hernandez-Marin, Maite Martinez-Aguillo, Maria Jose Lecumberri, Angela Fernandez de Lascoiti, Lucia Teijeira, Idoia Morilla, Ruth Vera, David Escors, Grazyna Kochan

**Affiliations:** 1 Navarrabiomed-Fundacion Miguel Servet, IdISNA, Irunlarrea 3, 31008 Pamplona, Navarra, Spain; ai.bocanegra.gondan@navarra.es (A.B.); miren.zuazo.ibarra@navarra.es (M.Z.-I.); hugo.arasanz.esteban@navarra.es (H.A.); mjgarciagranda@gmail.com (M.J.G.-G.); carlos.hernandez.saez@navarra.es (C.H.); maria.ibanez.vea@navarra.es (M.I.); 2 Department of Oncology, Complejo Hospitalario de Navarra, IdISNA, Irunlarrea 3, 31008 Pamplona, Navarra, Spain; gonzalo.fernandez.hinojal@navarra.es (G.F.-H.); oncomedica.hgugm@salud.madrid.org (B.H.-M.); maite.martinez.aguillo@navarra.es (M.M.-A.); mj.lecumberri.biurrun@navarra.es (M.J.L.); a.fdz.de@navarra.es (A.F.d.L.); lucia.teijeira.sanchez@navarra.es (L.T.); idoia.morilla.ruiz@navarra.es (I.M.); 3 Division of Infection and Immunity, University College London, 5 University Street, London WC1R 6JJ, UK

**Keywords:** PD-L1, biomarker, lung cancer, immunotherapy, immune checkpoint blockade

## Abstract

PD-L1 tumor expression is a widely used biomarker for patient stratification in PD-L1/PD-1 blockade anticancer therapies, particularly for lung cancer. However, the reliability of this marker is still under debate. Moreover, PD-L1 is widely expressed by many immune cell types, and little is known on the relevance of systemic PD-L1^+^ cells for responses to immune checkpoint blockade. We present two clinical cases of patients with non-small cell lung cancer (NSCLC) and PD-L1-negative tumors treated with atezolizumab that showed either objective responses or progression. These patients showed major differences in the distribution of PD-L1 expression within systemic immune cells. Based on these results, an exploratory study was carried out with 32 cases of NSCLC patients undergoing PD-L1/PD-1 blockade therapies, to compare PD-L1 expression profiles and their relationships with clinical outcomes. Significant differences in the percentage of PD-L1^+^ CD11b^+^ myeloid cell populations were found between objective responders and non-responders. Patients with percentages of PD-L1^+^ CD11b^+^ cells above 30% before the start of immunotherapy showed response rates of 50%, and 70% when combined with memory CD4 T cell profiling. These findings indicate that quantification of systemic PD-L1^+^ myeloid cell subsets could provide a simple biomarker for patient stratification, even if biopsies are scored as PD-L1 null.

## 1. Introduction

Lung cancer is the first (24.8%) and second (14.2%) cause of death by cancer in men and women, respectively [1]. Around 85–90% of them are diagnosed as non-small cell lung cancer (NSCLC), which is poorly responsive to conventional treatments. Approximately 30–40% of total cases are diagnosed at advanced stages (III and IV), leaving less treatment options and lower survival rates.

The introduction of immune checkpoint inhibitors is rapidly changing the therapeutic approaches in oncology, especially the blockade of PD-L1/PD-1 interactions. PD-1 and PD-L1 form a signaling axis that inhibits T cell anti-tumor activities but also protects cancer cells against cytotoxic agents [2,3,4]. PD-L1/PD-1 blockade is demonstrating good overall efficacies for the treatment of many tumor types, and it is currently used for the treatment of NSCLC [5], melanoma [6,7], head and neck squamous cell carcinoma (HNSCC) [8,9], renal cell carcinoma [10,11], urothelial carcinoma [12], gastric cancer [13], microsatellite instability-high (MSI-H) cancers [14], and mismatch-repair deficiency [15].

As PD-L1 is frequently expressed by tumors to inhibit T cells and survive their cytotoxic activities, the quantification of PD-L1 tumor expression in biopsies has been used as a predictive biomarker of responses to anti-PD-L1/anti-PD-1 therapies. [16,17,18,19] However, the reliability of PD-L1 tumor expression is still under debate because the determination of cut-off expression values is still confusing. Several issues interfere with immunohistochemistry, such as tissue preparation, antibody clones, processing variability, primary versus metastatic biopsies, or staining of tumor versus immune cells [20]. As a consequence, major differences can be observed even in clinical trials.

Indeed, a review study in which PD-L1 tumor expression was assessed in NSCLC patients revealed high discrepancies from association of PD-L1 expression with better prognosis, worse prognosis, or no prognosis at all [21]. An important drawback is the frequent lack of biopsies in NSCLC patients, and PD-L1 expression from cytology samples can be rather unreliable. Liquid biopsy provides an alternative to immunohistochemistry, as it is easier to obtain and can be collected and analyzed repetitively during treatment.

PD-L1 is also highly expressed in circulating immune cell populations in different tumor types [22,23,24,25,26]. Indeed, PD-L1 can be expressed at high levels in dendritic cells [3] and myeloid-derived suppressor cells [27], where it plays a fundamental regulatory role in T cell activation during antigen presentation, or regulation of excessive inflammation [28,29]. Hence, it could be possible that the baseline distribution of PD-L1 expression in systemically circulating immune cells might indeed contribute to clinical responses to PD-L1/PD-1 blockade therapies.

In the present study, we studied two very similar NSCLC clinical cases treated with atezolizumab with differing clinical outcomes. Both patients were classified as having PD-L1 negative tumors by immunohistochemistry, but one responded objectively to therapy while the other progressed quickly. These patients showed different PD-L1 expression profiles in peripheral blood cells, which prompted us to assess PD-L1 expression and distribution on circulating immune cell populations before the start of immunotherapy in an exploratory study with 31 patients with advanced NSCLC. Our results showed significant differences between responders and non-responders and explored the clinical implication of PD-L1 within these cell populations in response to immunotherapy.

## 2. Results

### 2.1. Case Study 1. Objective Responder

Patient 1 (LA058) is a 47-year-old male with a personal history of diabetes mellitus type 1, aortic valve insufficiency, and a smoking history of 20 cigarette packages per year. He was diagnosed in September 2010 with a stage IIIA (cT4N0M0) lung adenocarcinoma, with the primary tumor at the aorto-pulmonary window. He showed a nearly complete response to cisplatin/etoposide chemotherapy concurrently with radiotherapy. Seven months later, he relapsed with an upper right lobe (URL) metastasis and regrowth of the primary mass. The disease stabilized after six cycles of carboplatin/pemetrexed therapy. Progression was detected three months later, and the patient started systemic treatments with docetaxel-bevacizumab (stable disease after six cycles), then erlotinib (progression at three months), then gemcitabine (stable disease but progressing after six months), and finally vinorelbine (progression after three cycles with a new suprarenal lesion). Right suparrenalectomy was performed and sterotactic body radiation therapy on the URL node was administered in April 2015.

In April 2016, a paravertebral mass and a contralateral upper left lobe metastasis (ULL) were detected with slow progression. The patient exhibited good performance (ECOG0), absence of symptoms, and slow growth of the disease.

**Figure 1:**
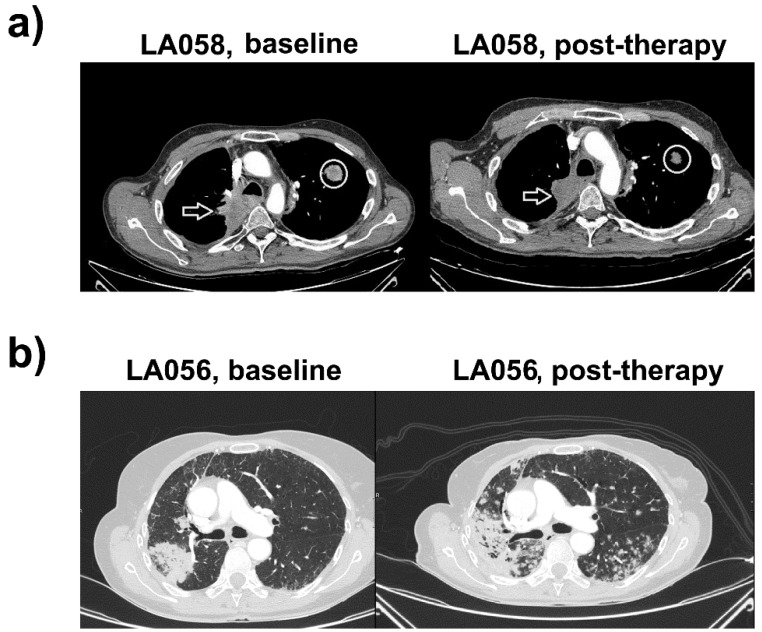
Clinical responses for tumor PD-L1-negative patients LA058 and LA056 treated with atezolizumab. (**a**) Left, CT images of LA058 before the start of immunotherapy. Right, CT images of LA058 after atezolizumab, demonstrating objective clinical responses, with evident tumor shrinkage. Arrows indicate a paravertebral upper right lobe mass with mediastinal invasion and posterolateral wall of the trachea. The circle indicates a contralateral metastatic node in the upper left lobe. Both lesions show a marked morphological decrease compatible with a partial response. (**b**) Left, CT evaluation of the LA056 patient. A tumoral relapse in the site of previous surgical intervention is evident. Right, CT following atezolizumab treatment. Tumor progression with bilateral pulmonary nodes and increase of the lower right lobe (LRL) mass, consistent with fast progressive disease.

In April 2018, he presented progressive dyspnea and asthenia, with progression of the paravertebral mass and the ULL node (Figure 1a). PD-L1 expression in a tumor sample obtained by bronchoscopy was negative, and the status of ROS1 and ALK rearrangements and EGFR mutation were non-informative. Treatment with 1200 mg q21d atezolizumab (anti-PD-L1) was started, without significant side-effects and evident clinical improvement. The right paravertebral mass and the ULL node showed shrinkage after four cycles of therapy, and absence of new lesions, compatible with a partial response (Figure 1a). He is currently under treatment with adequate tolerance to treatment.

### 2.2. Case Study 2. Progressor

Patient 2 (LA056) is a 64-year-old woman with a smoking history of 40 cigarette packages per year and hypercholesterolemia, with long-lasting bronchopneumonia and a suspicious mass in the Lower Right Lobe (LLR). PET-CT scan uncovered a 7-cm-wide lesion in the LLR with high metabolic activity and another lesion in the Middle Right Lobe (Figure 1b). Results from fine-needle aspiration suggested an adenocarcinoma. Bilobectomy of the lower right lobe and the middle lobe was performed together with hilar-mediastinal lymphadenectomy. The patient was diagnosed with pT4N0M0 (stage IIIA) lung adenocarcinoma with ipsilateral nodes and lack of nodal involvement. Subsequent study of molecular markers in cancer cells (ALK, ROS1, EFGR mutations) were negative. PD-L1 expression in the tumor was null.

The patient underwent four cycles of adjuvant chemotherapy with intravenous cisplatin (80 mg/m^2^) on day one, plus vinorelbine (25 mg/m^2^) on days one and eight q21d, presenting grade two diarrhea. The patient presented progression with bilateral pulmonary nodes and tumor relapse at the previous surgical site. Palliative chemotherapy with pemetrexed (500 mg/m^2^ q21d) was initiated, achieving stabilization. After 10 cycles of treatment, CT scans showed an increase in the number and size of the pulmonary nodes (Figure 1b).

Treatment with atezolizumab was initiated. The patient presented fever, cough, and progressive dyspnea after six cycles. Increases in the number and mass of contralateral pulmonary nodes without implication of bacterial or fungal infections was observed, consistent with progressive disease (Figure 1b).

### 2.3. Distribution of PD-L1 Expression in Systemic Immune Cells from the Two Clinical Cases

**Figure 2:**
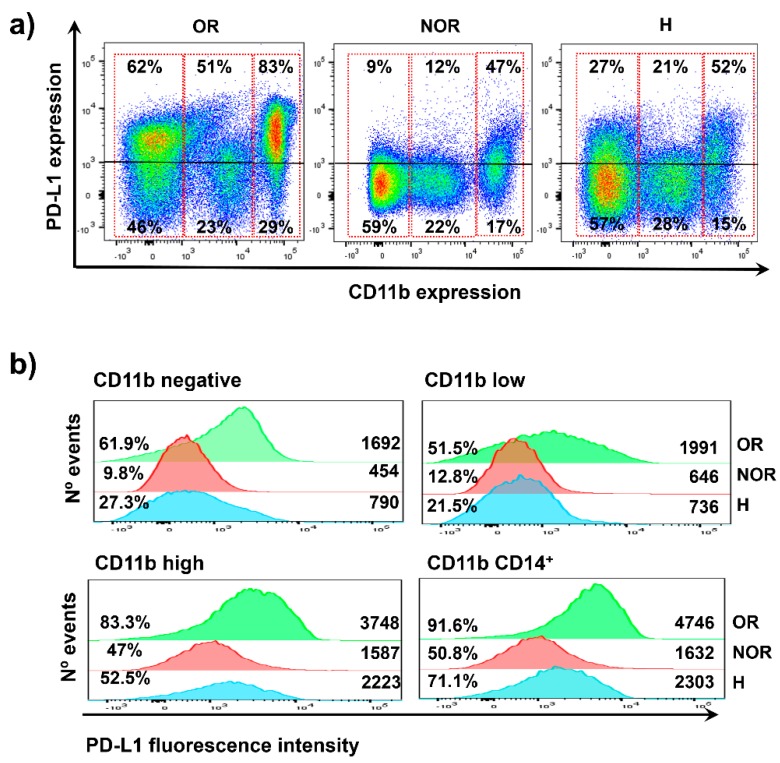
PD-L1 expression within systemically circulating immune cells in patients LA058 and LA056. (**a**) Flow cytometry density plots of PD-L1 expression in systemic immune cells for the objective responder patient LA058 (OR, left); for the progressor patient LA056 (non-objective responder, NOR, center); and for an age-matched healthy donor (H) as a control. (**b**) Flow cytometry histograms representing PD-L1 expression levels in systemic immune cells from the indicated subjects (OR, NOR, and H denote objective responder, non-objective responder, and healthy donor, respectively). The percentage of PD-L1 positive cells and mean fluorescent intensities are indicated within the graphs.

Both clinical cases were similar and were scored as tumor PD-L1 negative. However, one of them responded efficaciously to anti-PD-L1 immunotherapy (atezolizumab) and the other did not. We then set out to investigate if there were systemic differences in PD-L1 expression that could explain the differential clinical outcomes. We retrospectively analyzed high-dimensional flow cytometry data that was performed in baseline fresh peripheral blood mononuclear cell (PBMC) fractions from these patients using a collection of selected markers (materials and methods) for the identification of the main immune cell lineages (myeloid cells, T, NK, and NKT cells, B cells) together with expression of relevant markers, including PD-L1. To find out if there were differences in PD-L1 expression in peripheral immune subsets that could have influenced the opposite clinical outcomes to anti-PD-L1 therapy, we plotted PD-L1 expression in flow cytometry density plots using FlowJo (Figure 2a). PD-L1 was detected at variable levels in the majority of immune cells, with highest expression in CD11b^+^ subsets. Then, the expression of surface PD-L1 was then analyzed as a function of CD11b by flow cytometry density plots (Figure 2a). This immune cell marker is highly expressed within the myeloid populations. In agreement with SPADE data, systemic immune cells could be broadly divided into CD11b^high^, CD11b^low^, and CD11b^negative^ subsets (Figure 2). CD11b^high^ cells englobe monocytes, monocytic myeloid-derived suppressor cells (MDSCs), and neutrophils. CD11b^low^ corresponds to dendritic cells (DCs), granulocytic MDSCs, some T cells, and NK cells. CD11b-negative subsets contain most of the T lymphocytes, B cells, and plasmacytoid DCs. A sample from an age-matched healthy donor was included as a reference. Interestingly, the objective responder exhibited very high percentages of PD-L1-expressing cells in CD11b^negative^ and CD11b^high^ immune cell types, but not in CD11b^low^ cells (Figure 2a). The data also showed that PD-L1 expression levels were also superior (Figure 2b). These results indicated that major differences in the distribution of PD-L1 expression within major lineages could contribute to objective clinical responses, even if tumors were identified as PD-L1 negative.

### 2.4. The Baseline Percentage of Systemic PD-L1^+^ CD11b^+^ Cells Correlates with Objective Clinical Responses

The results from the two clinical cases prompted us to analyze additional retrospective high-dimensional flow cytometry data in an exploratory cohort of 32 NSCLC patients under treatment with PD-L1/PD-1 blockade therapies. Patient characteristics are summarized in Table 1.

**Figure 3:**
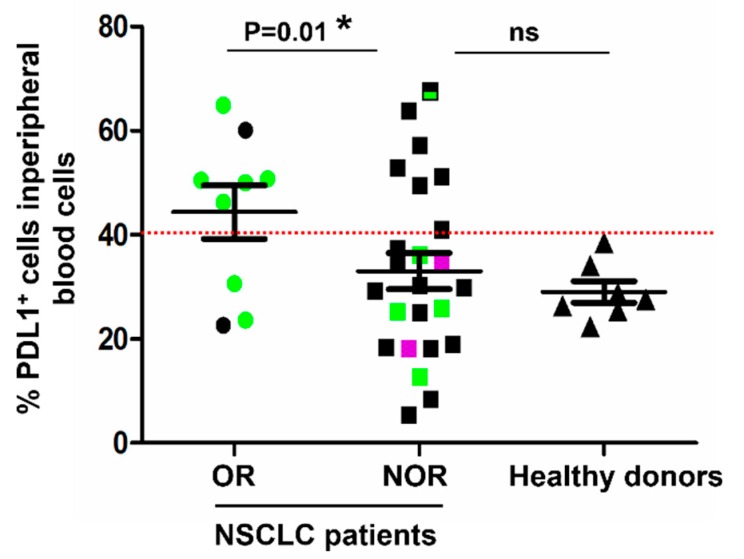
Quantification of PD-L1^+^ cell subsets in systemic immune cells and correlation with clinical responses. Dot plot graph representing the percentage of PD-L1^+^ cells within total systemic immune cells quantified from fresh peripheral blood samples before the start of immunotherapies, in objective responders (OR, N = 9), non-responders (NOR, N = 24), and healthy donors (N = 7). Relevant statistical comparisons are shown within the graph, by the exact test of Fisher. In green, patients with >40% circulating memory CD4 T cells. In purple, patients with stable disease. In black, patients with <40% circulating memory CD4 T cells. The dotted red line indicates the cut-off value used to test the association of the percentage of PD-L1^+^ T cells with clinical responses.

To find out if PD-L1 expression within total systemically circulating immune cells could be a good baseline indicator of clinical responses, patients were grouped into objective responders and non-objective responders. A sample of age-matched healthy donors was also included in the analyses, and the percentage of PD-L1^+^ cells was plotted. Interestingly, there was a significant association (*p* = 0.01) between patients with a high (>30%) systemic percentage of PD-L1^+^ cells before the start of immunotherapies and objective clinical responses after therapy administration (Figure 3). In a previous study, we characterized the contribution of systemic central memory and effector memory CD4 T cells to clinical responses to immunotherapy [30]. We observed that patients with more that 40% of baseline memory CD4 T cells exhibited response rates of 50%. Therefore, we tested the overlap of these patients with PD-L1 positivity (Figure 3). Interestingly, patients with high percentages of memory CD4 T cells and low percentages (<40%) of PD-L1^+^ cells within total systemic immune cells did not respond objectively to PD-L1/PD-1 blockade therapies.

**Figure 4:**
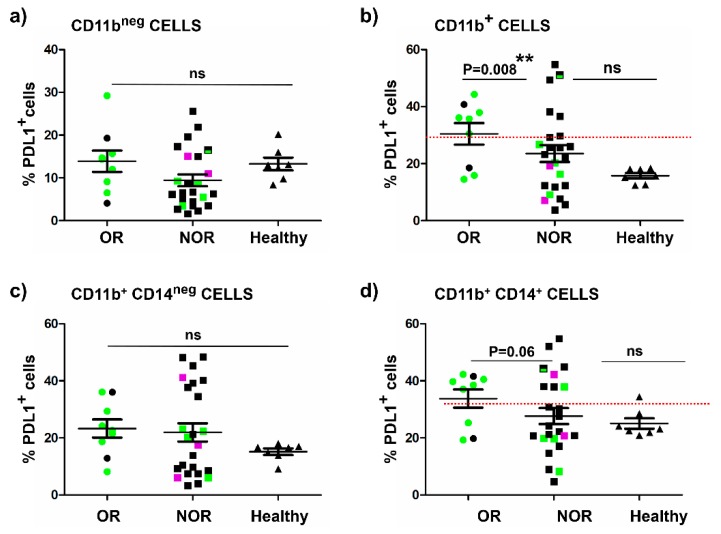
Quantification of PD-L1^+^ cell subsets in different compartments of immune cell types in peripheral blood and correlation with clinical responses. (**a**) Dot plot graph representing the percentage of PD-L1^+^ cells within systemic CD11b^negative^ subsets quantified from fresh peripheral blood samples before the start of immunotherapies, in objective responders (OR, N = 9), non-responders (NOR, N = 24), and healthy donors (N = 7). (**b**) Within CD11b^+^ cell subsets. (**c**) Within CD11b^+^ CD14^negative^ subsets. (**d**) Within CD11b+ CD14+ subsets. Relevant statistical comparisons are indicated within each graph, by the Fisher’s exact test, considering as cut-off values the indicated with horizontal red dotted lines. Means ± standard deviations are shown within the dot plots. Green, patients with >40% of systemic memory CD4 T cells; Black, patients with <40% of systemic memory CD4 T cells; Violet, patients with stable disease.

To find out if these global differences in PD-L1 expression occurred within CD11b^negative^ immune cells as observed between the two clinical cases (Figure 2), the percentage of PD-L1^+^ cells within CD11b^negative^ cells was plotted in objective responders, non-responders, and a small cohort of healthy donors. Interestingly, there were no differences between PD-L1 expression in CD11b^negative^ cells and clinical responses (Figure 4a). In contrast, a very significant association was found between a high systemic percentage of PD-L1^+^ CD11b^+^ with objective responders (Figure 4b). CD11b^+^ cells can be further divided into CD14^negative^ and CD14^+^ (monocytic) subsets. We evaluated PD-L1 expression within monocytic subsets and its relationship with objective responses. Interestingly, there was a tendency for objective responders to have more than 30% of systemic CD11b^+^ CD14^+^ cells expressing PD-L1, although the differences were at the verge of statistical significance by the Fisher’s association test (*p* = 0.06) (Figure 4c). No association was found with CD11b+ CD14^negative^ cells PD-L1+ cells (Figure 4d). Again, combining PD-L1 expression with CD4 T cell stratification showed that patients with high content (more than 40%) of memory CD4 T cells who did not respond to treatment were also characterized by low percentages of PD-L1^+^ CD11b^+^ cells.

Overall, these results suggested that a high percentage of systemically circulating PD-L1^+^ CD11b^+^ immune cells before the start of immunotherapies could be a good indicator of objective clinical responses to PD-L1/PD-1 blockade therapies. Its combination together with quantification of circulating memory CD4 T cells (Table 2) can help to identify patients with a high probability of response.

## 3. Discussion

Response rates to PD-L1/PD-1 blockade immunotherapy for the treatment of lung cancer account for 20–30% of patients. Nevertheless, these responses still double those achieved with classical therapies. This increase in number of responders and overall survival rates is leading to a wider application of immunotherapy.

PD-L1 tumor expression, either by cancer cells or immune infiltrates, is currently used as a stratifying clinical factor. Hence, patients with >5% PD-L1 tumor expression usually show clinical benefits from PD-L1/PD-1 blockade therapies. This underlines the importance of accurate determination of PD-L1 expression levels. However, there is some controversy on the usefulness of PD-L1 tumor quantification. Importantly, biopsies are not always available, especially for NSCLC patients. PD-L1 expression is also quantified in circulating tumor cells as a substitute for biopsying the lesions.

In our clinical practice, we have observed that patients scored as tumor PD-L1 null can still respond to anti-PD-L1 therapies. An example of two divergent cases was shown in the present study, treated with atezolizumab (PD-L1-binding antibody). These results prompted us to study the implication of PD-L1 expression by other cells. As atezolizumab is administered systemically, we evaluated the distribution of PD-L1 expression in major systemic immune cell lineages by high-dimensional flow cytometry in a small exploratory study with NSCLC patients undergoing PD-L1/PD-1 blockade. Interestingly, we observed a significant correlation between the percentage of PD-L1^+^ CD11b^+^ myeloid cells and objective clinical responses. We found that patients exhibiting a baseline percentage >30% had a response rate of 50%. We can discard age as a factor affecting our conclusions, since the cohort of patients was homogeneous in age (majority were over 60 years old), and the healthy donors were age-matched. These results are in agreement with previous reports indicating that a relatively high baseline percentage of circulating CD14^+^ HLA-DR^+^ monocytes could be a predictor of responses to anti-PD-1 treatment [31]. However, in this latter study, the status of PD-L1 positivity within myeloid cells was not taken into consideration, and analyses were performed by CyTOFF coupled to machine-learning for identification of predictive biomarkers. Currently, these techniques cannot be translated into routine clinical practice; however, our approach is straightforward and only requires standard flow cytometry for quantification of PD-L1^+^ CD11b^+^ subsets.

In a previous work, we also showed a significant correlation between circulating highly differentiated memory CD4 T cells and objective clinical responses. Patients with high percentages of these memory subsets within the CD4 cell population also had about 50% response rates [30,32]. Interestingly, patients with high memory CD4 T cells that also had lower percentages of PD-L1^+^ CD11b^+^ cells did not show objective responses. These results suggest that combining key systemic cellular parameters could be used to stratify patients with a high probability of responding to PD-L1/PD-1 blockade.

Our results also strongly suggest a direct role for PD-L1-expressing CD11b^+^ myeloid cells in clinical responses. It is likely that PD-L1 blockade in these cells may enhance their antigen presentation capacities, and together with memory CD4 T cells trigger strong anti-tumor activities.

We are currently expanding these analyses during immunotherapy treatments to evaluate the dynamic changes of these myeloid populations in responders and progressors.

## 4. Materials and Methods

### 4.1. Study Design

The Ethics Committee of the Hospital Complex of Navarre approved the current study (Comité Ético de Investigación Clínica, CEIC, Department of Health of the Government of Navarre; Pabellón de Docencia. Irunlarrea 3, 31008, Pamplona, Navarre, Spain. Reference: Pyto 2017/91, approved on 17 October 2017). The experimental design followed the principles of the Declaration of Helsinki and Good Clinical Practice guidelines. Each participant provided written informed consent. Samples were collected by the Blood and Tissue Bank of Navarre, Health Department of Navarre, Spain. Thirty-one patients with non-squamous or squamous NSCLC were recruited (Table 1) who had progressed to first line chemotherapy or chemo-radiotherapy, or were given first-line immunotherapies. Eligible patients who received PD-1/PD-L1 blockade immunotherapy were at least 18 years of age (Table 1).

Nivolumab (BMS, New York, USA), pembrolizumab (MSD, New Jersey, USA), or atezolizumab (Roche, Basel, Switzerland) was administered to patients as part of their standard therapies [5,33,34]. Four milliliter samples of peripheral blood were retrieved before the start of immunotherapy. PBMCs were isolated as we described before [35] and cells analyzed by flow cytometry following standard protocols. Tumor responses were evaluated according to the criteria defined by RECIST 1.1 [36] and also by the Immune-Related Response Criteria [37].

### 4.2. Flow Cytometry and High-Dimensional Analyses by SPADE

PBMC isolation and processing and analyses by flow cytometry were performed as described previously [35]. Briefly, 10 mL of blood specimen was obtained as venipuncture, collected into sterile ethylenediaminetetraacetic acid (EDTA) tubes on the day of the start of therapy and prior to treatment. Samples were transferred to the research laboratory within 1 h. Subsequently, PBMCs were purified by Ficoll-Plaque (GE, Chicago, IL, USA) centrifugation and labelled for analysis with mouse monoclonal antibodies specific for CD3, CD4, CD8, CD19, CD56, CD54, HLA-DR, and PD-L1 (Biolegend, San Diego, CA, USA), CD11b, CD14, CD15, CD33 (Miltenyi, Bergisch Gladbach, Germany). 

### 4.3. Data Collection and Statistics

The percentage of the appropriate immune cell populations was quantified with Flowjo (FlowJo LLC, Ashland, OR, USA). Data between group were prespecified to be analyzed by Student t-tests or ANOVA if normally distributed, or by the U of Mann–Whitney or Krukall–Wallis if not normally distributed or for data with intrinsic variability. Fisher’s exact test was used to assess the association of the baseline values of immune cells with clinical responses. Statistical tests were performed with the GraphPad Prism 5 (GraphPad Software, San Diego, CA, USA) statistical package.

## 5. Conclusions

Our results also strongly suggest a direct role for PD-L1-expressing CD11b^+^ myeloid cells in clinical responses. Therefore apart from PDL1 quantification in the tumor it would be important to analyse PDL1 expression in systemic blood cell subsets before therapy application.

## Figures and Tables

**Table 1:** Baseline characteristics of patients involved in the study.

Variable	All Patients (N = 32)
**Sex**	
Female	12
Male	20
**Age**	
<60	10
≥60	22
**Histology**	
Squamous	10
Non-Squamous	21
**Immunotherapy treatment**	
Pembrolizumab	9
Nivolumab	15
Atezolizumab	8
**PDL1 status**	
0%	8
1–4%	3
5–49%	8
≥50%	7
Undetermined	6
**Mutation status**	
No	30
EGFR	1
ROS1	1
**Smoking status**	
Smoker	27
Non-smoker	5
**Treatment line**	
1st	5
2nd	20
3rd	5
4th or higher	2
**Previous systemic therapies (previous 3 months)**	
Platinum-based therapy	12
Non-platinum based therapy	8
No	12
**ECOG**	
0–1	25
2–4	7
Undetermined	0
**GRImScore**	
0–1	15
2–3	7
Undetermined	9
**Liver metastases**	
No	23
Yes	9
**Number of sites involved**	
≤2	9
≥3	23
**CD4 THD Profiling**	
G1 profile	14
G2 profile	18
**Responses**	
Partial response	10
Progression disease	19
Stable disease	3

**Table 2:** Patient stratification according to PD-L1 expression in myeloid cells combined with memory CD4 T cell profile and associated response rates.

Target Patient Population	Response Rate
**PD-L1 CD11b^+^ >30%**	
Memory CD4 T cells >40%	70% (5/7)
Memory CD4 T cells <40%	15% (1/6)
**PD-L1 CD11b^+^ <30%**	
Memory CD4 T cells >40%	33% (2/6)
Memory CD4 T cells <40%	6% (1/13)

## Abbreviations

CD	Cluster of differentiation
DC	Dendritic cell
ECOG	Eastern cooperative oncology group
HNSCC	Head and neck squamous cell carcinoma
MDSC	Myeloid-derived suppressor cell
NSCLC	Non-small cell lung cancer
OR	Objective responder
PBMC	Peripheral blood mononuclear cell
PD-L1	Programmed death-1 ligand 1
PD-1	Programmed death-1
NOR	Non-objective responder
ULL	Upper left lobe
URL	Upper right lobe
